# The Role of Bioactive Lipids in Stem Cell Mobilization and Homing: Novel Therapeutics for Myocardial Ischemia

**DOI:** 10.1155/2014/653543

**Published:** 2014-01-06

**Authors:** Yuri M. Klyachkin, Anush V. Karapetyan, Mariusz Z. Ratajczak, Ahmed Abdel-Latif

**Affiliations:** ^1^Gill Heart Institute and Division of Cardiovascular Medicine, University of Kentucky and Lexington VA Medical Center, Lexington, KY 40536-020, USA; ^2^Stem Cell Biology Institute, James Graham Brown Cancer Center, University of Louisville, Louisville, KY 40202, USA; ^3^Department of Physiology, Pomeranian Medical University, Szczecin, Poland

## Abstract

Despite significant advances in medical therapy and interventional strategies, the prognosis of millions of patients with acute myocardial infarction (AMI) and ischemic heart disease (IHD) remains poor. Currently, short of heart transplantation with all of its inherit limitations, there are no available treatment strategies that replace the infarcted myocardium. It is now well established that cardiomyocytes undergo continuous renewal, with contribution from bone marrow (BM)-derived stem/progenitor cells (SPCs). This phenomenon is upregulated during AMI by initiating multiple innate reparatory mechanisms through which BMSPCs are mobilized towards the ischemic myocardium and contribute to myocardial regeneration. While a role for the SDF-1/CXCR4 axis in retention of BMSPCs in bone marrow is undisputed, its exclusive role in their mobilization and homing to a highly proteolytic microenvironment, such as the ischemic/infarcted myocardium, is currently being challenged. Recent evidence suggests a pivotal role for bioactive lipids in the mobilization of BMSPCs at the early stages following AMI and their homing towards ischemic myocardium. This review highlights the recent advances in our understanding of the mechanisms of stem cell mobilization, provides newer evidence implicating bioactive lipids in BMSPC mobilization and differentiation, and discusses their potential as therapeutic agents in the treatment of IHD.

## 1. Introduction: Ischemic Heart Disease

Ischemic heart disease (IHD), which includes heart failure induced by myocardial infarction (MI), is the single most prevalent cause of morbidity and mortality worldwide. Currently, IHD caused 1 of every 6 deaths in the United States, and despite the significant advancements in medical and revascularization therapies, the prognosis of millions of patients with ischemic heart disease remains poor [1]. IHD results from the partial or complete interruption of oxygenated blood supply to the heart muscle primarily due to an occlusion of a coronary artery. The resulting ischemia causes myocardial cell death and, if left untreated, results in extensive tissue damage. While heart transplantation is a viable therapy to replace the infarcted myocardium it is still plagued by limited availability of donors, peri- and postprocedural complications, side effects of immunosuppressive therapies, and overall less than optimal patient prognosis. Until recently, the notion that MI-damaged myocardium could regenerate was non-existent. This review will examine breakthroughs in cardiac stem cell biology and recent advances in cell-based therapies to treat ischemic myocardium.

## 2. The Role BM-Derived Cells in Continuous Renewal of Cardiomyocytes

Until a decade ago, it was believed that the human heart was a postmitotic organ that is not capable of self-renewal, and therefore the MI-damaged myocardium could not be regenerated. However, this dogma has been refuted by multiple groups. The study by Quaini et al., investigating the chimerism of sex-mismatched transplanted heart, presented early evidence for myocardial regeneration by demonstrating active renewal of all three major cell lines in human hearts. The number of recipient-originated cardiomyocytes, vascular smooth muscle cells, and endothelial cells increased significantly in hearts from female donors that were transplanted into male recipients. Furthermore, these primitive cells, which originated in the bone marrow (BM), expressed stem cell antigens including c-kit, MDR1, and Sca-1. Interestingly, a fraction of these cells were Y-chromosome-positive, providing direct evidence that these cells translocated from the host to the myocardium of the grafted heart. Moreover, migration of these primitive cell populations to the grafted heart resulted in their loss of stem-cell markers, active proliferation, and acquisition of the mature phenotype followed by cell colonization and de novo formation of myocytes, coronary arterioles, and capillaries [2]. To address the question of BM origin of chimeric myocytes, the follow-up investigation analyzed hearts of patients who have undergone gender-mismatched BM transplantation. The key findings suggested that BM acts as a source of extracardiac progenitor cells contributing to cardiomyocyte formation and accounts for at least part of the cell chimerism observed in other studies. Interestingly, the potential origin and phenotype of marrow myocyte precursors included lineage-restricted mesenchymal, hematopoietic, and multipotent adult progenitor cells [3]. Together, these data established human bone marrow as a source of bone marrow stem/progenitor cells (BMSPCs) capable of de novo cardiomyocyte formation and possibly repair. However, the mechanisms governing the mobilization of BM cells from their niches to the myocardium are poorly understood. The literature suggests that the magnitude of this phenomenon is significant replacing at least half of the adult cardiomyocytes during normal physiological aging [4]. Anversa's group demonstrated higher chimerism with physiological aging and in heart failure [5]. In this study, the human adult heart is capable of replacing its entire population of cardiomyocytes, endothelial cells and fibroblasts 6–8 times during normal life span and under physiological conditions. The chimerism of cardiomyocytes is age dependent and is also influenced with pathological conditions such as heart failure [5] and ischemic injury [6].

Hematopoietic stem/progenitor cells (HSPCs) escape their BM niche in response to chemotactic gradients and can be detected in the PB under steady state conditions [7]. Numerous factors have been shown to be responsible for HSPC mobilization including strenuous exercise [8], tissue, or organ injury (including ischemic cardiac events) [9, 10] and may significantly increase in circulation after administration of pharmacological agents [11, 12]. BMSPCs have multifaceted roles in an adult organism most importantly being involved in lymphohematopoiesis [7] and immune surveillance [13]. Initially, multiple groups have demonstrated that the gradient of chemotactic stromal derived factor-1 (SDF-1) is the major determining factor of BMSPCs' destination [14, 15]. Since BMSPCs express the SDF-1 receptor-CXCR4 and SDF-1 is expressed by osteoblasts and fibroblasts in the BM microenvironment, it is undisputed that the resulting SDF-1-CXCR4 interaction results in BMSPCs' retention in the BM niches [15]. Furthermore, BMSPCs express very late antigen-4 (VLA-4, also known as *α*_4_ *β*_1_-integrin) while the cells in the BM microenvironment express its ligand, vascular adhesion molecule-1 (also known as CD106), and further contribute to BMSPCs BM retention [16–18].

Lately, however, the chemokine-exclusive paradigm was challenged by numerous observations supporting SDF-1-CXCR4-independent homing and mobilization of BMSPCs. Specifically, it was shown by numerous groups that the plasma SDF-1 level does not always correlate with mobilization of BMSPCs [19–22]. While some studies have observed chemotaxis of BMSPCs to an increased SDF-1 gradient, the SDF-1 was administered at supraphysiological concentrations (100–300 ng/mL) [23, 24], which is about 100 times higher than the SDF-1 concentrations measured in human or murine biological fluids [25]. Also, SDF-1 upregulation was observed in tissues injured by hypoxia, and similar upregulation takes place in the BM microenvironment during conditioning for transplantation by radiochemotherapy or after administration of pharmacological agents that promote mobilization of BMSPCs (such as G-CSF or a CXCR4 antagonist AMD3100) [18, 26–28]. Ironically, these conditions induce upregulation of several proteolytic enzymes released by BM cells, such as metalloproteinase 2 (MMP-2), MMP-9, cathepsin G, and neutrophil elastase, thereby proteolytically inactivating SDF-1 and CXCR4 and neutralizing chemotactic activity of SDF-1 towards BMSPCs [26, 29, 30]. It is important to note that this proteolytic environment would promote HSPC mobilization by decreasing SDF-1-CXCR4-mediated retention (as well as attenuating VLA-4-CD106 interaction); however SDF-1 homing would be impaired due to enhanced proteolytic degradation of SDF-1 [18, 26, 31]. Together, these observations imply that other possibly protease-resistant chemoattractants are involved in HSPC mobilization in order to make up for the deficiency of the SDF-1 gradient between the BM and PB. The above findings directed the investigation towards proteolysis-resistant sphingolipids, specifically sphingophospholipids (sphingosine 1-phosphate and ceramide 1-phosphate), which were shown to be potent chemoattractants for BMSPCs.

## 3. Sphingolipids and Stem Cell Signaling

Sphingolipids are a class of lipids consisting of a backbone composed of sphingoid bases and an amino alcohol sphingosine [32]. Initially, they were believed to be sheathing nerves and the interest in their research remained confined to a small group of scientists. As the evidence for pathophysiological importance of sphingolipids grew, so did their research field. As of today sphingolipids are shown to be involved in a wide variety of biological responses in a diversity of cell types including stimulation of cell proliferation, inhibition of apoptosis, and regulation of cell shape and cell motility [33–36].

**Figure 1:**
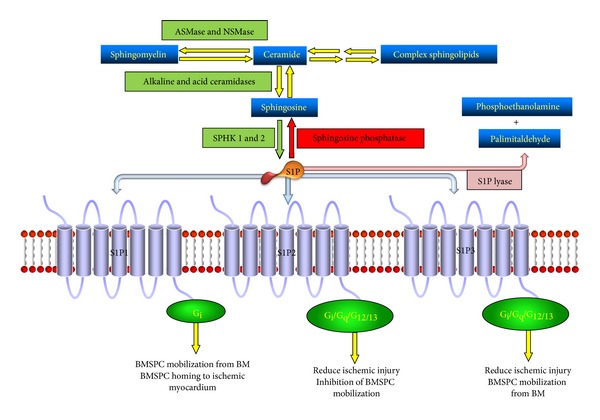
Sphingosine 1-phosphate (S1P) metabolism and signaling in bone marrow stem and progenitor cells (BMSPCs). Interconversion of membrane sphingolipids and final phosphorylation of sphingosine by SPHKs results in formation of S1P which signals through S1P1, S1P2 and S1P3 receptors in the BMSPCs. These three receptors activate a distinct set of pathways through G_i_, G_q_, or G_12/13_ proteins which results in BMSPC mobilization from the bone marrow niches (S1P1 and S1P3); inhibition of BMSPC mobilization from the bone marrow (S1P2); and BMSPC homing to ischemic myocardium (S1P1).

Sphingolipids are important structural components of cell membranes and are derived from ceramide, the proverbial “core” of sphingolipid metabolism. Ceramide can be deacylated to sphingosine which is then phosphorylated by sphingosine kinases (SPHK1 or SPHK2) to yield sphingosine 1-phosphate (S1P) (Figure 1). Both transcripts of SPHK1 and SPHK2 are subject to alternative splicing resulting in multiple isoforms for each kinase [37]. Transgenic mouse studies have demonstrated partial redundancy of SPHK1 and SPHK2 since SPHK1^−/−^ or SPHK2^−/−^ mice were phenotypically normal while elimination of both genes resulted in embryonic death [38, 39] indicating that S1P is produced exclusively by SPHKs *in vivo*. Ceramide 1-phosphate (C1P) can be generated by phosphorylation of ceramide (N-acyl sphingosine) by ceramide kinase [40]. Both S1P and C1P have limited half-lives and their levels are kept in check by numerous enzymes. S1P is irreversibly degraded by S1P lyase and is also regulated by lipid phosphate phosphatases (LPP1–3) and S1P-specific phosphatases (SPP1 and SPP2) [41–45], and C1P is regulated by LPP1–3 [41, 45]. The major source of plasma S1P are red blood cells, activated platelets, albumin, high-density lipoproteins, and extracellular SPHK1 derived from vascular endothelial cells [33, 46, 47], while the primary contribution to C1P plasma levels comes from intracellular C1P which has been released or “leaked” from damaged cells [48].

Upon their release, both S1P and C1P interact with a variety of G protein-coupled seven-transmembrane receptors. There are 5 S1P receptor subtypes (S1P_1–5_) that are widely expressed throughout mammalian tissues. S1P_4_ and S1P_5_ are expressed and function in the immune and nervous system, respectively, S1P_1–3_ are most abundant throughout the cardiovascular system and are expressed on BMSPCs. S1P_1_ is coupled exclusively via G_i_ to Ras-MAP kinase, phosphoinositide (PI) 3-kinase-Akt pathway and phospholipase C pathway. S1P_2_ and S1P_3_ are coupled to multiple G proteins, such as G_q_, G_12/13_ and G_i_ to activate phospholipase C pathway and Rho pathway [34–36, 49]. The signaling cascade activated by S1P binding to either S1P_1_ or S1P_3_ is responsible for HSPC migration [13, 50, 51]. Activation of S1P_2_ and, however, yields an opposite effect, negatively regulating HSPC mobilization [52]. While the receptor for C1P is yet to be identified, its signaling is sensitive to pertussis toxin, thereby implicating a G_i_ protein coupled receptor [53, 54].

## 4. Sphingosine 1-Phosphate Chemoattracts BMSPCs

Once S1P receptors were discovered on BMSPCs, they were immediately characterized as G protein-coupled seven-transmembrane receptor thereby placing them in the same class as chemokine receptors. This observation raised one important question: can S1P act as a direct chemoattractant for BMSPCs? Initially, Seitz et al. demonstrated a dose-dependent chemotactic effect of S1P on human HSPCs in a modified Boyden chamber assay [19]. It is possible that polarizing doses of S1P promote signaling through the S1P_2_ receptor, which in contrast to S1P_1_, inhibits HSPC chemotaxis [21]. Subsequent studies established that the gradient of S1P between BM and PB is a major determining factor in HSPCs egress. While SDF-1 still has a significant role in HSPCs mobilization, it was demonstrated that plasma derived from normal and mobilized PB strongly chemoattracts murine BM HSPCs independent of plasma SDF-1 levels [21]. This was especially evident when removal of lipids from plasma by charcoal stripping abolished HSPCs chemotaxis but did not affect responsiveness towards SDF-1 [21]. Ratajczak et al. further showed that steady state S1P plasma levels create a gradient favoring HSPCs egress from the BM. As previously described, HSPCs are actively retained in BM via SDF-1-CXCR4 and VLA4-V-CAM1 interactions. Ratajczak et al. corroborated the significance of S1P in HSPCs chemotaxis by demonstrating that disruption of these interactions via CXCR4 antagonist AMD3100 or triggering a proteolytic environment in the BM would release HSPCs form their niches and therefore free them to follow the bioactive lipids' gradient to PB.

Furthermore, Ratajczak et al. showed that a robust innate immune response during G-CSF mobilization is responsible for increased plasma S1P levels. G-CSF is currently the most frequently used mobilizing agent that efficiently mobilizes BMSPCs after a few consecutive daily injections [55]. It has been established that G-CSF triggers complement complex activation which stimulates granulocytes to release proteolytic enzymes, thereby perturbing SDF-1-CXCR4/VLA-4-VCAM1 interactions in BM niches and facilitating HSPCs release [27]. Remarkably, the lasting effect of G-CSF promotes CC activation and formation of the membrane attack complex (MAC) that was shown to interact with erythrocytes [56]. While erythrocytes serve as the major reservoir of S1P in the PB [20, 57], they are highly protected from MAC by CD59 and decay-accelerating factor (DAF) receptors [58]. However, Ratajczak et al. demonstrated that expression of these receptors on erythrocytes does not give complete protection from activated MAC since G-CSF-induced MAC exposure resulted in plasma S1P levels sufficient for HSPCs egress [21].

While it has been established that S1P is responsible for HSPC trafficking, the mechanism to explain this regulation is still under investigation. Recent evidence suggests that SDF-1 and S1P work synergistically to facilitate migration of primitive murine progenitor cells out of the BM [59]. Further *in vitro* studies on immature human CD34+ cells demonstrated that S1P_1_ upregulation decreases their chemotactic activity towards SDF-1 due to reduced cell surface expression of CXCR4 suggesting a potential interaction between S1P and SDF-1 [60]. These observations were recently corroborated by Golan et al. showing that short-term inhibition of S1P/S1P_1_ axis during steady state conditions or during CXCR4 inhibition (via AMD3100 administration) caused reduction of SDF-1 in the plasma [61]. Interestingly, generation of reactive oxygen species (ROS) via S1P_1_ signaling were also implicated in HSPCs mobilization through the release of SDF-1 [62]. Since previous studies showed that ROS inhibition reduces SDF-1 secretion during AMD3100-induced mobilization [63], it was hypothesized that ROS signaling might also contribute to SDF-1 secretion. Indeed it was demonstrated that ROS signaling induced SDF-1 secretion thereby facilitating HSPCs egress [61].

S1P-SDF-1 interaction in HSPCs egress was further demonstrated with the help of FTY720, a potent S1P_1_ desensitizing agent which causes S1P receptor internalization [64]. Interestingly, administration of FTY720 for 24 hours resulted in increased plasma SDF-1 levels but had no effect on HSPCs egress. FTY720 treatment did reduce BM ROS signaling, due to S1P_1_ downregulation, again pointing out the requirement of S1P_1_ signaling in HSPC egress. Furthermore, mice that were treated with BM-specific S1P lyase inhibitor 4-deoxypyridoxine (DOP) [44] had increased BM ROS levels and decreased HSPC egress [61]. Together, these observations suggest that the increased concentrations of S1P and SDF-1 in the BM negatively affect HSPC egress, further highlighting the fact that both S1P and SDF-1 levels must be tightly regulated for balanced HSPCs mobilization.

**Figure 2:**
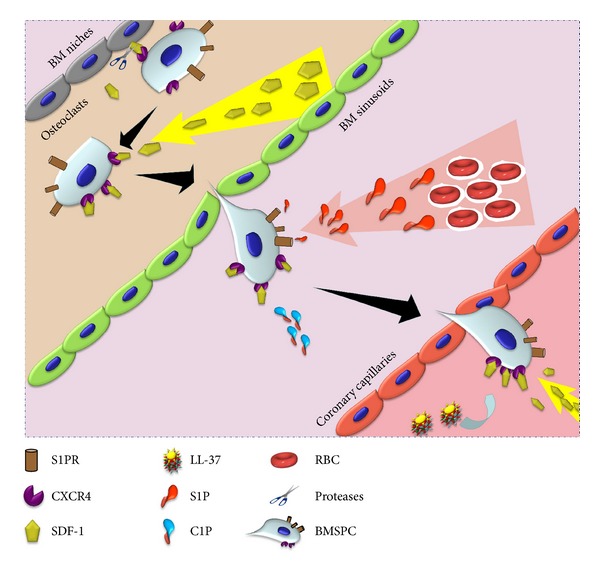
Sequence of events in BMSPC mobilization from the BM towards ischemic myocardium during MI. Acute MI initiates an inflammatory response resulting in release of proteases (by granulocytes and osteoclasts) in the BM which proteolytically inactivate the SDF-1 CXCR4 interaction between BM osteoclasts and BMSPCS. The now mobilized BMSPCs follow an increasing SDF-1 and bioactive lipid (S1P and C1P) gradient to exit the BM niches into the PB. Acute inflammation also promotes the release of cathelicidins (LL-37) which facilitate clustering of CXCR4 into lipid rafts thereby increasing their sensitivity towards lower levels of circulating SDF-1. Together, the increased sensitivity towards SDF-1 and bioactive lipid gradients facilitate BMSPC homing towards ischemic myocardium.

While bioactive lipids such as S1P and C1P are powerful mobilizers of BMSPCs, their role in BMSPCs' mobilization and homing to ischemic myocardium is not well understood. The role of other chemoattractants in BMSPCs homing to a hostile environment such as the infarcted myocardium is also unclear. We recently examined the role of bioactive lipids, complement, and antimicrobial peptides in BMSPC homing during MI. Our data shows elevated level of S1P and C1P in the plasma of MI patients shortly after the onset of MI [65]. Increased S1P and C1P levels were correlated with elevated numbers of circulating BMSPCs suggesting a role of bioactive lipids in BMSPCs mobilization post-MI [65]. Our speculations were corroborated by a modified Boyden chamber assay (chemotaxis assay) where we observed increased BMSPCs' chemotaxis towards plasma isolated from patients at peak BMSPCs mobilization. Moreover, this migration was selectively blocked by VPC23019, a specific S1P_1_ antagonist, further implicating S1P as a potent BMSPCs chemoattractant during MI [65]. As previously described, MI induces a potent proteolytic environment in which numerous enzymes such as metalloproteinases and proteases irreversibly degrade potent BMSPCs chemoattractants such as SDF-1. Recent evidence suggests a role for antimicrobial protein cathelicidin LL-37 in sensitizing BMSPCs towards significantly lower levels of SDF-1 [66]. LL-37 sensitizes BMSPCs by incorporating CXCR4 into the lipid rafts thereby augmenting CXCR4 signaling. Most importantly, we observed LL-37 overexpression following MI in cardiac tissues as well as cardiac fibroblasts. Furthermore, we implemented the chemotaxis assay to confirm that priming BMSPCs with LL-37 from patients with MI increases their mobilization to low, yet physiological, levels of SDF-1 (2 ng/mL) [65]. Taken together, our findings highlight the importance of bioactive lipids and innate immunity in the mobilization and homing of BMSPCs to the ischemic myocardium (Figure 2).

## 5. Mobilization of BMSPC Populations during MI

The BM cell populations commonly referred to as BMSPCs is a heterogeneous population of cells consisting of hematopoietic stem cells (HSCs), endothelial progenitor cells (EPCs), mesenchymal stromal cells (MSCs), and pluripotent very small embryonic-like cells (VSELs). Acute myocardial infarction (MI) initiates a systemic inflammatory response that stimulates a signaling cascade that results in egress of BMSPCs from the BM. At the onset of MI, BMSPSs are mobilized following the gradient of a multitude of previously described chemoattractants, including bioactive phospholipids, kinins, chemokines, cytokines, growth factors, and the complement cascade [67–72]. Several studies have demonstrated the chimerism of cardiomyocytes, a process that is maintained, at least in part, by BM-derived stem cells [2, 3, 73, 74]. Innate cardiomyocyte renewal is an effective process that replaces up to 45% of the adult cardiomyocytes during the normal lifespan [4] suggesting that development of cardiac cell based therapies is feasible and essential to treat MI-damaged myocardium. The heterogeneity of the BM cell populations could be responsible for the differential response to the multitude of chemoattractants.

## 6. Fate of Adults Stem Cells after MI

### 6.1. Biology of Infarcted Myocardium

However, the question still remains what is the fate of BMSPCs once they reach the ischemic myocardium? The fate of BMSPCs is ultimately determined by the nature of the myocardial microenvironment. The onset of ischemic injury and subsequent reperfusion results in a robust proinflammatory state with elevated levels of locally activated complement [75–77] and ROS [78]. The induction of ROS and subsequent cytokine cascade contributes to rapid neutrophil infiltration of the infarct region, with neutrophil levels peaking between 24 and 72 hours after MI [76, 79, 80]. Furthermore, neutrophils attracted to the ischemic myocardium release proteolytic enzymes and additional ROS which may cause collateral damage to the infiltrating stem cells. It is clear that the above described mechanisms as well as the lack of a good blood supply to the infarct region make the myocardial microenvironment early on post-MI unsuitable for stem cell arrival. However, as soon as 5 days after MI the acute inflammatory response subsides, and angiogenesis, the major factor in infarct healing, begins to take place [81, 82]. Thus, around this time, when the acute inflammatory response has decreased and the infarct site is being vascularized, stem cells might find the right environment to attach and proliferate. This window of opportunity is limited, however, due to evidence of extensive scar formation as soon as 2 weeks after MI, which would hinder stem cell nesting [82–84]. Clinical studies' findings are in agreement with these pathological findings and temporal trends. REPAIR-AMI study demonstrated that the beneficial effects of BM derived mononuclear cells were most evident when the cells were transplanted ≥5 days after the acute myocardial injury [85].

### 6.2. Therapeutic Effects of Adult Stem Cells after MI

Once the stem cells do survive their journey to the infarcted myocardium after MI, their ultimate contribution to myocardial repair is still unclear. Since adult myocardium has very limited potential for self-regeneration, the stem cells may contribute to ischemic myocardium repair via various paracrine mechanisms or differentiation into endothelium and/or cardiomyocytes. There is ample evidence supporting the hypothesis that paracrine mechanisms mediated by factors released by the adult stem cells play an essential role in myocardial repair after stem cell mobilization following MI. Numerous groups have shown that adult stem cells, and especially mesenchymal stem cells (MSCs; described above), secrete a broad range of chemokines, cytokines, and growth factors that are potentially involved in cardiac repair [86]. Interestingly, hypoxia at the site of injury in conjunction with stem cell administration further stimulates production of these factors which includes hepatocyte growth factor (HGF), vascular endothelial growth factor (VEGF), insulin-like growth factor (IGF)-I, basic fibroblast growth factor (bFGF), and adrenomedullin [87, 88]. The paracrine benefits were further corroborated by administering conditioned medium (CM) from adult stem cells and comparing those effects to actual stem cell therapy [89–91]. These paracrine effects of BM derived cells extend to other populations such as cKit+ cells and VSELs, thereby contributing to regeneration of ischemic myocardium [92]. The authors demonstrated that the improvement in cardiac functions were disproportionate to the rate of differentiation of BM derived cells suggesting that various factors secreted by these cells can explain the majority of beneficial effects. We observed similar findings with VSELs in an animal model of acute ischemic injury [93].

Differentiation of adult stem cells into cardiomyocytes and their subsequent contribution to post-MI repair has been extensively investigated for more than a decade. Initial *in vitro* studies were able to isolate adult stem cells from either BM or adipose tissue and through various culture conditions, induce their differentiation into beating cells exhibiting cardiomyocyte morphology and physiology [94–103]. Although recent *in vivo* studies have revealed that heart cells are generated in adult mammals during normal homeostasis as well as post-MI, the frequency of generation and the source of new heart cells remain unclear. Some studies suggest a high rate of stem cell differentiation into cardiomyocytes [104]. Other studies suggest that new cardiomyocytes are derived from the division of preexisting cardiomyocytes at a very slow rate [4, 105, 106]. Most recently Senyo et al. showed that the genesis of cardiomyocytes occurs at a low rate by the division of preexisting cardiomyocytes during normal ageing and this process was markedly increased in areas adjacent to myocardial injury [107]. However, the evidence presented herein accents the fact that adult stem cells contribute to myocardial repair by a wide array of effects including paracrine mechanisms, differentiating into functional tissues (cardiac or endothelial), as well as other diverse therapeutic features, to preserve yet undamaged cells and contribute to endogenous creation of new functional tissue.

### 6.3. The Role of S1P in Adult Stem Cell Differentiation

While evidence suggests that S1P is involved in BMSPC mobilization, the possibility still remains that bioactive lipid signaling may contribute to the ultimate fate of BMSPCs post-MI.

**Figure 3:**
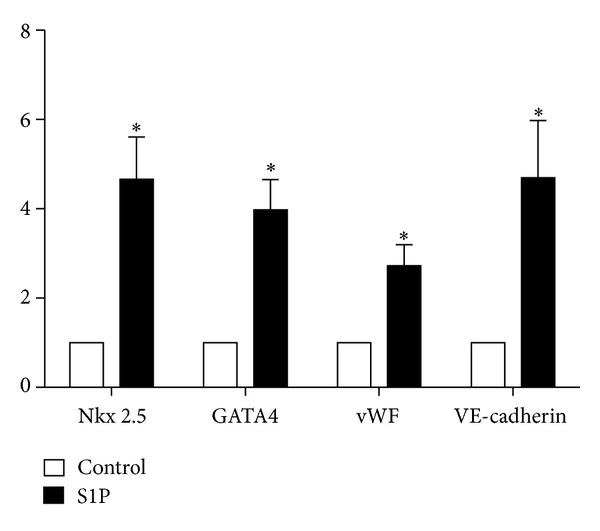
Effect of S1P on expression of cardiac and endothelial factors in BMSPCs. Bar graphs showing the mRNA expression of cardiac markers, Nkx2.5 and GATA4, and endothelial markers, vWF and VE-cadherin in cells following 48 hours of treatment with 250 nM S1P. Cells incubated with S1P were enriched in these genes compared to controls (*P < 0.05).

**Figure 4:**
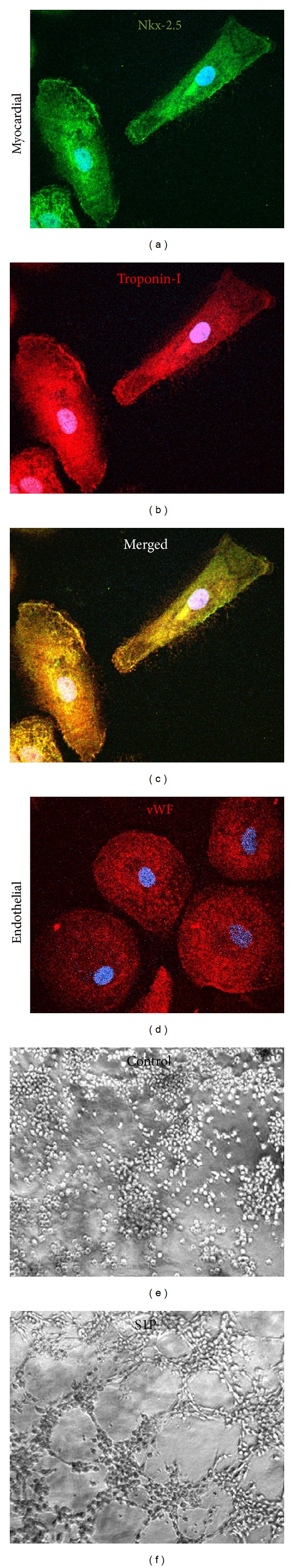
Effect of S1P on cardiac and endothelial protein expression in BMSPCs. Representative confocal and matrigel assay images of BMSPCs cultured for 4 and 2 weeks in culture medium supplemented with 250 nM of S1P. Panels (a)–(c) depict elongated cells indicative of cardiomyocyte morphology. (a) Visualization of early myocardial protein expression (GATA4 and Nkx-2.5—green). (b) Staining of late myocardial protein expression (myosin heavy chain and Troponin—red). (c) Overlay of (a) and (b), depicting colocalization of myocardial protein expression in the cytoplasm and cardiac transcription factors around the nucleus. Panel (d) depicts round cells indicative of endothelial morphology and visualization of endothelial protein expression (VWF, PDGFr*α*, and PDGFr*β*—red). All cells were stained with DAPI to visualize the nuclei (blue). (e) and (f) matrigel assay images demonstrate the capillary-like structure formation by BMSPCs cultured in S1P medium.

**Figure 5:**
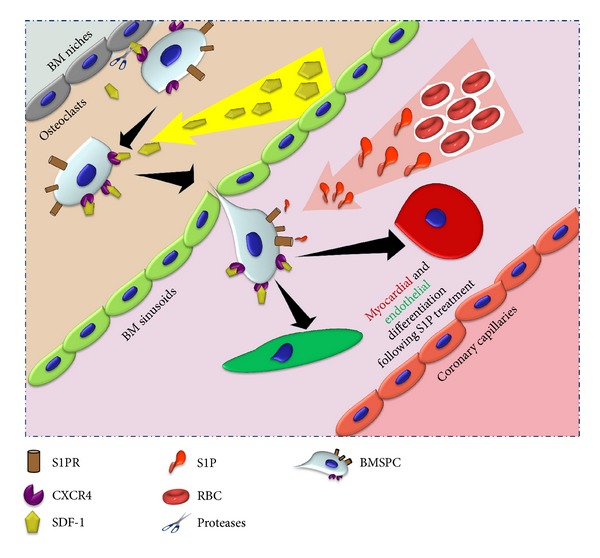
Proposed BM-derived stem cell differentiation scheme in the presence of S1P. We propose that following myocardial infarction the role of S1P is twofold: mobilization of BMSPCs from their BM niches as well as promoting their subsequent differentiation into myocardial and endothelial lineages, thereby further aiding in myocardial repair following ischemic injury.

Since S1P is a potent intracellular second messenger it has been implicated in numerous physiological processes including vasculogenesis. Interestingly, recent evidence has also demonstrated that S1P has promoted embryonic and neural stem cell differentiation, proliferation, and maintenance [108–110]. Recently, Zhao et al. demonstrated that S1P drove differentiation of human umbilical mesenchymal stem cells (HUMSCs) into cells exhibiting cardiomyocyte-like morphology and physiology, and ultimately the formation of cell sheets from HUMSCs derived cardiomyocytes [111]. This study was the first of its kind to demonstrate that S1P potentiates differentiation of HUMSCs towards functional cardiomyocytes. Furthermore, the engineered cell sheets provide potential for generating clinically applicable myocardial tissues. These newly discovered therapeutic effects of S1P prompted us to extend the work of Zhao et al. by assessing the ability of S1P to initiate differentiation of BMSPCs into cardiomyocytes and vascular cells. Indeed, incubation of BMSPCs with 250 nM of S1P resulted in initiation of cardiac and endothelial expression after 48 hours in cell culture.  Figure 3 demonstrates the increased expression of cardiac transcription factors (Nkx-2.5 and GATA4) as well as endothelial genes (vWF and VE-cadherin). Following our results indicating the increased expression of early cardiac and endothelial genes with S1P incubation, we proceeded with examining the expression of functional cardiac and endothelial proteins in BMSPCs cultured for 3-4 weeks in medium supplemented with 250 nM of S1P. BMSPCs expressed morphology suggestive of both cardiac (elongated) and endothelial (rounded) at 4 weeks of culture (Figure 4). Immunohistochemical examination revealed the expression of various cardiac proteins such as troponin and cardiac specific myosin heavy chain in the elongated cells. These proteins were expressed in the cytoplasm (Figures 4(b) and 4(c)). We also observed remnants of cardiac transcription factors such as Nkx-2.5 and GATA4 around the nucleus (Figures 4(a) and 4(c)). Similarly, rounded cells with endothelial morphology were found to have endothelial proteins such as vWF, VE-cadherin, PDGFr*α*, and PDGFr*β* in the cytoplasm suggestive of endothelial lineage differentiation (Figure 4(d)). In parallel, BMSPCs exposed to S1P demonstrated functional endothelial lineage commitment as demonstrated by 10-fold higher tube formation in matrigel assays (*P* < 0.05) (Figures 4(e) and 4(f)). Taken together, our results suggests that S1P may play a role in the differentiation of BM-derived stem cells. This is of clinical importance given our recent findings that confirm the elevation of S1P level in the plasma and its potential role in BMSPCs mobilization following acute myocardial infarction [65]. We propose that following myocardial infarction and the role of S1P is twofold: mobilization of BMSPCs from their BM niches as well as promoting their subsequent differentiation into myocardial and endothelial lineages thereby further aiding in myocardial repair following ischemic injury (Figure 5).

Multiple new therapies that modulate the plasma levels of S1P or its receptors' expression are approved by the FDA and can be utilized in improving the mobilization and differentiation of BMSPCs in myocardial ischemia in future myocardial regenerative studies. Similarly, priming BM-derived cells with LL-37 can be used to improve their homing to the ischemic myocardium and thus overcome a major hurdle in stem cell regenerative myocardial therapies. We are currently examining both strategies in our laboratory to improve the mobilization and homing of BMSPCs to the ischemic myocardium.

## 7. Conclusion

The emergence of bioactive lipids (S1P and C1P) as significant players in the trafficking of BMSPCs has added a new dimension to our understanding of BMSPC biology. Available literature and our findings highlight the importance of bioactive lipids in the mobilization and homing of BMSPCs to the ischemic myocardium. In conjunction with our data about their role in stem cell mobilization and homing, it appears that bioactive lipids have an additional role in promoting BMSPC differentiation and proliferation towards cardiac or endothelial tissue lineage. The field of stem cell-based myocardial regeneration still faces multiple challenges such as the appropriate stem cell population, timing of therapy, and the route of administration. Numerous strategies are currently being explored to improve stem cell delivery and retention to the ischemic myocardium following acute injury and in the setting of chronic ischemic heart disease. Our and others recent data provide evidence that innate immunity (cathelicidins and the complement cascade) contribute to BMSPC homing by modulating the sensitivity of BMSPC surface receptors, specifically CXCR4. Therefore, future studies utilizing those, and similar, agents can improve the yield of stem cell therapy in patients who are in dire need for regenerative therapies.
